# Children consider others’ need and reputation in costly sharing decisions

**DOI:** 10.1038/s41598-025-91648-y

**Published:** 2025-02-27

**Authors:** Kirsten H. Blakey

**Affiliations:** 1 Philosophy, Faculty of Arts and Humanities, University of Stirling, Stirling, UK; 2 Psychology, Faculty of Natural Sciences, University of Stirling, Stirling, UK; 3 Psychology, University of Toronto, Mississauga, ON Canada

**Keywords:** Costly sharing, Indirect reciprocity, Fairness, Social decision-making, Prosocial behaviour, Dictator game, Human behaviour, Evolution

## Abstract

Children’s sharing decisions are shaped by recipient characteristics such as need and reputation, yet studies often focus on one characteristic at a time. This research examines how combinations of recipient characteristics impact costly sharing decisions among 3- to 9-year-old children (*N* = 186). Children were informed about the material need (needy or not needy) and reputation (sharing or not sharing) of potential recipients before having the opportunity to share stickers with them. Results indicated that sharing was higher when the recipient was needy and increased more when the recipient had a reputation for sharing. Children shared over half of their stickers with a needy, sharing recipient, and less than half with a not needy, not sharing recipient. Children shared equally with recipients who were needy and not sharing or not needy and sharing, suggesting no preference for either characteristic. To explore the emotional benefits of sharing, children rated their own and the recipient’s mood before and after sharing, showing a greater increase in ratings of the recipient’s mood when more resources were shared. These findings suggest that children consider multiple recipient characteristics in their sharing decisions, demonstrating altruism toward those in need and indirectly reciprocating past sharing based on reputation.

## Introduction

Unlike other species, humans commonly engage in prosocial exchanges with non-kin and strangers, even those they are unlikely to meet again. These behaviours—proposed to promote cooperation^1^—entail helping or sharing with others without direct benefit to the donors^2,3^. Such exchanges are observed cross-culturally^4–7^ and from early in infancy^8–10^, though different prosocial behaviours manifest at different ages. For example, 18-month-olds reliably help others, non-costly sharing typically emerges in the preschool years and increases with age^8,11–13^, and altruistic costly sharing develops later still. The later development of costly sharing suggests it may be more cognitively demanding to decide whether to share with someone when doing so involves sacrificing personal gain or personal resources, while prioritising the interests of others.

Many factors have been proposed and shown to influence children’s decisions about how much to share^18–20^, who to share with^15,21,22^, and the potential benefits of sharing, including, social norms^23^, rectifying (social) inequality^24–26^, direct and indirect reciprocation^3,27–30^, and emotional gain^5,18,31^. Although such factors are rarely encountered in isolation and children are typically aware of multiple characteristics of the individuals they interact with, research has focused on studying these factors individually. There are limited exceptions, though these have focused on combinations of characteristics (e.g., inequality and social categories^24^ or generosity and kindness^27^) in non-costly resource allocation tasks. Therefore, the current study sought to explore age-related changes in how children consider combinations of recipient characteristics when making costly sharing decisions.

The most commonly used behavioural measure for investigating sharing decisions is the dictator game^32,33^, in which a ‘dictator’ can share resources between themselves and a recipient. Given the challenges of studying pairs of children in these roles, especially when looking at recipient characteristics, elements of the original dictator game have been modified^12,34,35^ to make it more suitable for use with children. Typically, resources are stickers or tokens which can be exchanged for prizes^11,12,36^, and the recipient is often represented by an image of a hypothetical or virtual character^22^, though similar results have been found using real peers^11^. Modified dictator games have been used to investigate salient recipient characteristics such as need and social categories, and those learned through firsthand experience or testimony such as generosity, merit, and reputation.

A substantial body of research has investigated how salient recipient characteristics, such as need (material or emotional)^8,22,24,25,37,38^, social categories (e.g., ingroup vs. outgroup)^16,26,35^, and relationships (e.g., friendship or status)^35,37,39^ influence children’s resource allocation and sharing decisions in costly and non-costly contexts. This research finds that children are more likely to share when the recipient is in need, belongs to their ingroup, has a closer relationship with them, or holds a higher status. Sharing in response to an individual’s need is particularly interesting because it can actively change the recipient’s situation, whereas the other factors mentioned tend to reflect biases or preferences regarding whom to share with, rather than achieving a functional outcome. For example, sharing more with a needy or disadvantaged individual may help level the playing field between individuals or between the sharer and the recipient. This can apply to both material and emotional needs, although research on the latter is limited. Regarding material need, age-related changes have been observed in children’s willingness to share more with a needy individual. While 3- to 4-year-old children share approximately equally between needy and non-needy individuals, or between themselves and a needy individual, by around 8 years old they share more (70–80%)^22,25^ with needy individuals, reflecting a shift in preference from equality to equity (rectifying inequity).

While sharing with needy individuals in pursuit of equality or equity provides some insight, it does not fully explain why children share with others they do not know or are unlikely to meet again. Cooperative strategies such as tit-for-tat sharing^40,41^—in which participants base their decisions on their previous experience with the recipient—are beneficial in cases of repeatedly interacting individuals. For example, 3-year-old’s share more with a partner who has previously been generous toward them^17^. The theory of direct reciprocity suggests that people are motivated to share in anticipation of having their generosity reciprocated or to reciprocate generosity shown to them. However, this does not explain why humans commonly share with individuals they are unlikely to meet again. In contrast, the theory of indirect reciprocity suggests that repeated interactions are unnecessary to elicit sharing behaviours^2,43^, rather, we also share based on someone’s prior sharing behaviour towards others. That is, sharing decisions are made according to individuals past behaviours, for example, their reputation for being generous, helpful, or cooperative with others.

Children strategically manage their reputation and make costly donations to maintain their image from around 5 years old^44^. Therefore, it is unsurprising that they also consider others’ reputation or merit when they share^22,45–47^. In many studies, information about a potential recipient’s reputation or merit is obtained firsthand through observing them interact with a third party^3,27^, yet we are often not able to observe such interactions and instead rely on receiving information about reputation through others testimony. One study that examined reputation delivered via testimony found that a recipients reputation predicted children’s sharing^22^ and there were age-related changes in sharing behaviour. Although 4-year-old children made more selfish allocations than 8-year-olds, they still rewarded previous moral behaviour (sharing or not pushing), despite a cost to themselves. While there was a difference in 4-year-olds’ sharing based on whether the recipient had a positive or negative reputation, the disparity was much greater for 8-year-olds. Specifically, 8-year-olds shared more when the recipient had a positive reputation and less when the recipient had a negative reputation compared to 4-year-olds. This evidence that children can take a potential recipient’s reputation into account in a costly sharing scenario—even when reputation information is obtained through testimony—raises the question of whether they can consider reputation when it is presented in combination with other recipient characteristics.

While recipient characteristics undoubtedly influence children’s sharing decisions, it is also important to explore the role of emotion in motivating prosocial behaviour^18,19^. Indeed, prosocial behaviour can help to reduce negative and foster positive emotions^48–50^. As previously noted, a recipient’s emotional need may influence sharing decisions in a similar way to material need. Yet to examine this, we need to understand whether children (1) recognise that their sharing decisions can impact a recipient’s emotions, and (2) how their own emotions may be affected by the same decisions. Research suggests that even from a very young age, children show signs of understanding how prosocial behaviour can positively impact others’ emotions. For example, children as young as 18 months demonstrate prosocial responses (helping, distracting, vocalising sympathy, sharing) to another’s sadness^51^, and 3- to 6-year-olds who better grasp the relation between generosity and happiness tend to share more^52^. However, findings on the relationship between children’s emotional states and their prosocial behaviour are mixed. Although some studies suggest that children’s emotions are predictive of their sharing behaviour^5,12,34,52,53^, others suggest that they are not^36^. Additionally, research has found that children display more happiness after engaging in costly giving^50^. Indeed, positive feelings promote and reward prosocial behaviour throughout development^49^. What remains unexplored is how children’s own emotion ratings change in response to their sharing decisions, and how they perceive the recipient’s emotions before and after their sharing choices, relative to their own. Limited evidence has been obtained from children’s self-reports, instead being derived from parental report, or observed via behavioural responses. However, children as young as 3 and 4 years old demonstrate self-evaluative emotion, and their prosocial choices may, at least in part, be driven by the emotion that they feel (happiness, guilt, pride)^52,54^. Regarding self-report measures, prosocial behaviour in 5- and 6-year-old’s—but not 3-year-olds—correlates with ratings of the recipients emotion, rather than their own^55^. This indicates that older children may be motivated by empathic concern for the recipient rather than their own emotions.

As outlined above, both the material need and reputation (for helping and generosity) of potential recipients influence children’s costly sharing decisions from as young as 4 years old, with age-related increases observed in their willingness to share. However, previous research into children’s sharing decisions have focused on the influence of individual recipient characteristics, despite them rarely being present in isolation. Oftentimes children are privy to information regarding multiple characteristics of those they interact with. Therefore, the primary aim of the current study was to examine how combinations of recipient characteristics impact costly sharing decisions among 3- to 9-year-old children (*N* = 186). A modified version of the dictator game was used to measure children’s sharing decisions after receiving information about an age and gender matched character’s material need (needy or not needy) and reputation for sharing (sharing or not sharing). In the first set of trials children only had information about the recipient’s need (*“hasn’t got any stickers”* or *“already has some stickers*”), while in the second set of trials with a new character they were informed about the recipient’s need—which matched that of the first character—*and* their reputation for sharing *(“shares with her friends”* or *“does not share with her friends”*). Therefore, in the second set of trials there were four combinations of recipient characteristics: (1) needy and sharing, (2) not needy and sharing, (3) needy and not sharing, and (4) not needy and not sharing. Children were assigned to one of these combinations. Children were expected to share more with recipients who were in need and those with a reputation for sharing, aligning with their preferences for equity and indirect reciprocation^2,43^, respectively. Given that younger children have previously shown preferences for sharing equally, while tending to engage in more equitable sharing, it was anticipated that there may be developmental differences in children’s approach to sharing. Additionally, older children were expected to demonstrate a greater difference in sharing behaviour based on the recipient’s reputation compared to younger children.

A second objective was to investigate whether children’s mood ratings—both for themselves and the recipient—influence sharing and how children’s sharing affects changes in ratings of their own mood and their perception of the recipients’ mood. To address this, children were asked to rate their own and the recipient’s mood using a mood chart with five faces ranging from very unhappy to very happy, before and after sharing with them. It was anticipated that sharing more stickers would lead to a greater increase in children’s ratings for both their own and the recipient’s mood.

## Results

### The effect of recipient characteristics on sharing

#### Need trials

**Fig. 1:**
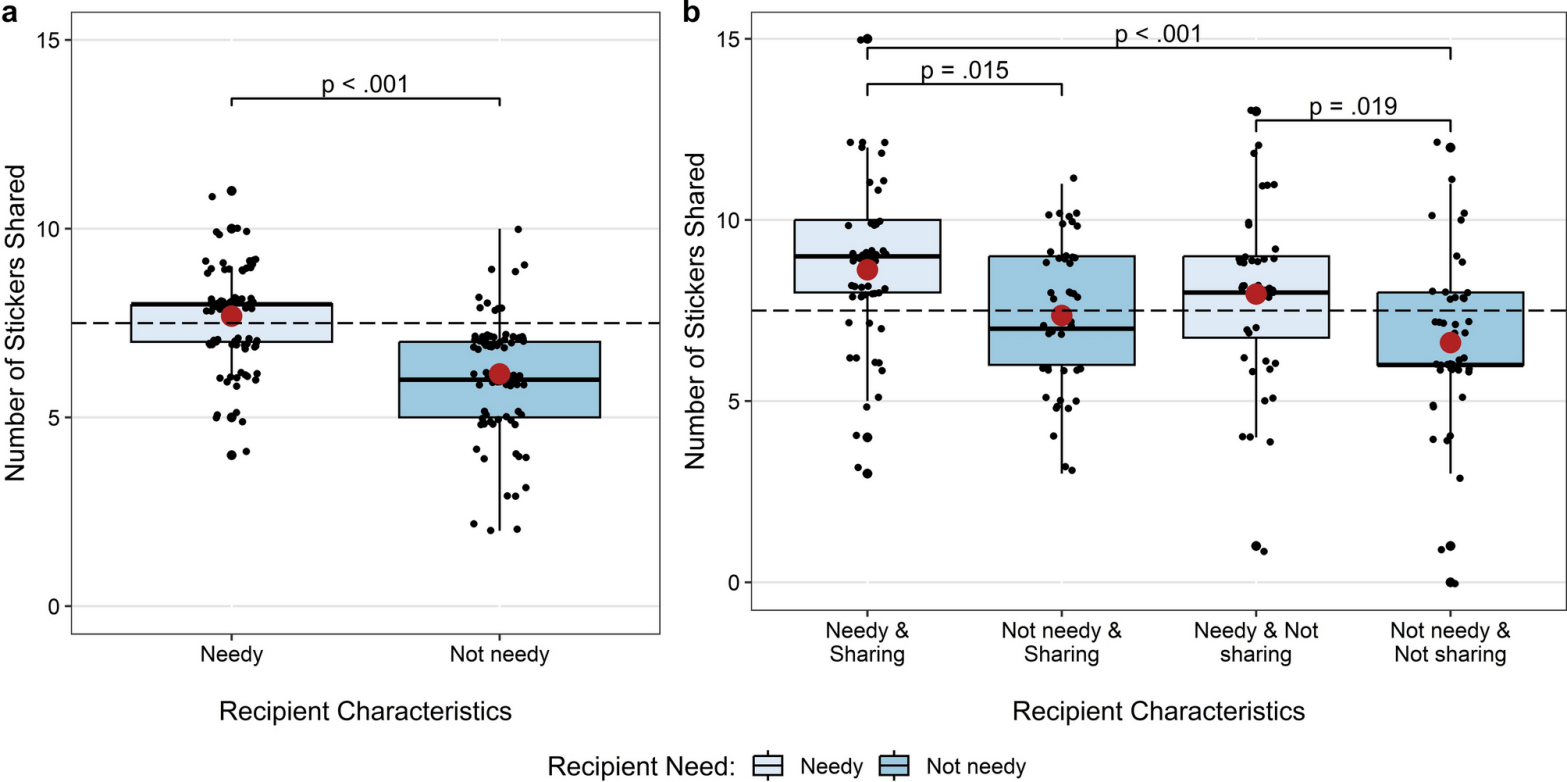
Mean number of stickers shared across the six trials (out of 15) in (**a**) the need trials and (**b**) the need and reputation trials. Small points show individual sharing scores, and large red points indicate group means. Brackets denote differences between groups. The dashed line represents equal sharing.

To assess whether children’s sharing choices were affected by recipients’ material need, a linear model was constructed with the number of stickers shared in the need trials as the dependent variable and recipient need, children’s age, and their interaction as fixed effects. This model was significantly better than the null model (*χ*^2^(3) = 114.9, *p* < 0.001). There was no evidence of an interaction between recipient need and age (*p* = 0.229) nor a main effect of age (*p* = 0.208). However, a significant main effect of recipient need (*b* = 0.77, *SE* = 0.10, *t*(182) = 7.63, *p* < 0.001, η^2^_*p*_ = 0.24) indicated that children shared significantly more with needy recipients (*M* = 7.68, *SD* = 1.23) than with not needy recipients (*M* = 6.15, *SD* = 1.53) (Fig. 1a).

The number of stickers shared was compared to equal sharing using one-sample *t*-tests. Children shared significantly less than half when the recipient was not needy, *t*(90) = –8.41, *p* < 0.001, but, shared equally when the recipient was needy (*p* = 0.148).

#### Change between need trials and need and reputation trials

To examine the impact of additional information about a recipient’s reputation for sharing on children’s sharing decisions, the change in the number of stickers shared between the need trials (taken as a baseline) and the need and reputation trials was calculated for each participant. Positive values indicate increased sharing in the need and reputation trials, while negative values reflect decreased sharing. A two-sample *t*-test indicated a significantly greater increase in children’s sharing scores when the recipient had a reputation for sharing (*M* = 1.05, *SD* = 2.00) compared to a reputation not sharing (*M* = 0.39, *SD* = 2.24), *t*(175.34) = 2.13, *p* = 0.035.

#### Need and reputation trials

A linear model was built to assess the influence of combinations of recipients’ need and reputation on children’s sharing, with the number of stickers shared as the dependent variable. The model included fixed effects of combination and age, and their interaction, and was significantly better than the null (*χ*^2^(7) = 155.8, *p* < 0.001). No interaction between combination and age was found (*p* ≥ 0.466), though sharing did increase significantly with age (*b* = 0.78, *SE* = 0.26, *t*(178) = 2.96, *p* = 0.003, η^2^_*p*_ = 0.05). There was a significant main effect of combination, *F*(3, 178) = 7.66, *p* < 0.001, η^2^_*p*_ = 0.11 (Fig. 1b). Post hoc pairwise comparisons revealed that children shared significantly more with the needy sharing recipient than the not needy sharing recipient (*b* = 1.35, *SE* = 0.45, *t*(178) = 3.03, *p* = 0.015) or the not needy not sharing recipient (*b* = 2.04, *SE* = 0.45, *t*(178) = 4.51, *p* < 0.001), and significantly more with the needy not sharing recipient over the not needy not sharing recipient (*b* = 1.38, *SE* = 0.47, *t*(178) = 2.95, *p* = 0.019). There were no significant differences between the two needy recipients (*p* = 0.467), the two not needy recipients (*p* = 0.438), or the needy not sharing and not needy sharing recipients (*p* = 0.444).

One-sample *t*-tests compared the number of stickers shared to sharing equally for each combination of recipient characteristics. Children shared significantly more than half with the needy sharing recipient (*M* = 8.63, *SD* = 2.24, *t*(50) = 3.59, *p* < 0.001), equally with the needy not sharing recipient (*M* = 7.95, *SD* = 2.36, *p* = 0.209) and not needy sharing recipient (*M* = 7.36, *SD* = 2.03, *p* = 0.642), and significantly less than half with the not needy not sharing recipient (*M* = 6.61, *SD* = 2.31, *t*(43) = –2.54, *p* = 0.015).

### The effect of mood rating on sharing

For each set of trials, an additional linear model was run to explore the influence of initial mood ratings on sharing. These models included additional fixed effects of children’s initial mood ratings for themselves and the recipient. Neither model showed any evidence of a significant effect of either mood rating (*p* ≥ 0.202). For full results see Supplementary Information.

### The effect of sharing on mood ratings

**Fig. 2:**
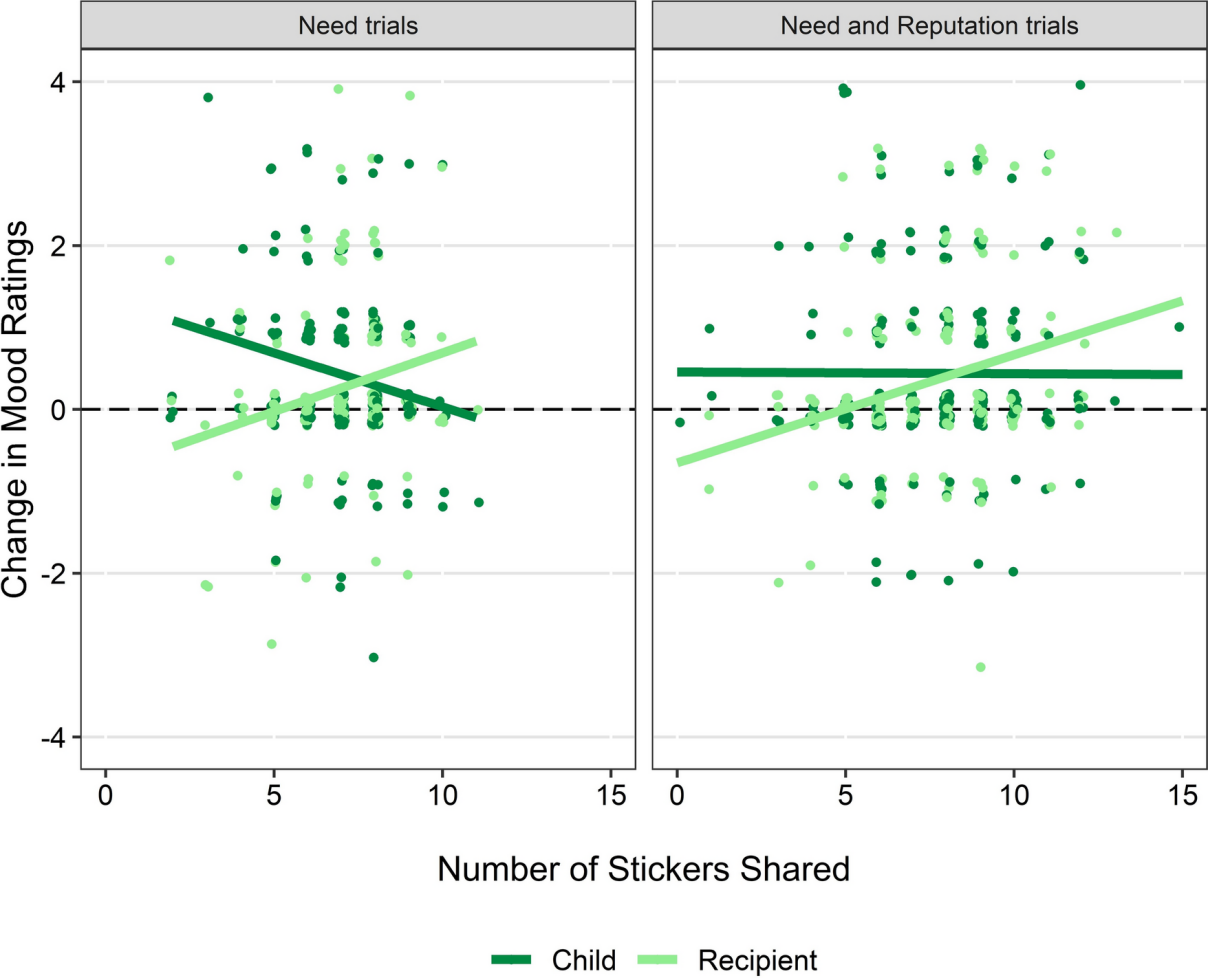
Change in children’s ratings of their own and the recipient’s mood ratings after sharing trials by number of stickers shared.

To examine how children’s sharing influenced their ratings of their own and the recipients’ mood, changes in their emotion ratings from before to after sharing were calculated separately for the need and need and reputation trials (Fig. 2). A LMM was then built for each set of trials with the change in mood rating as the dependent variable.

#### Need trials

The model included fixed effects of the number of stickers shared, recipient need, whose mood was being rated, their interactions, and children’s age, with participant ID as a random intercept. The model was significantly better than the null equivalent (*χ*^2^(8) = 29.21, *p* < 0.001), showing that the change in mood ratings was significantly greater when children were rating their own mood (*M* = 0.44, *SD* = 1.08) compared to the recipients’ mood (*M* = 0.25, *SD* = 0.99) (*b* = 0.94, *SE* = 0.26, *t*(186) = 3.56, *p* < 0.001) and that the change in mood ratings was significantly greater in older children (*b* = 0.20, *SE* = 0.90, *t*(186) = 2.23, *p* = 0.027). The model also revealed a significant two-way interaction between the number of stickers shared and whose mood was being rated (*b* = −0.12, *SE* = 0.04, *t*(186) = −3.33, *p* = 0.001). Post hoc *emtrends* analysis revealed that as the number of stickers shared increased, the change in children’s ratings of the recipients’ mood also increased, while the change in their ratings of their own mood decreased. This difference in trends was significant (*b* = −0.24, *SE* = 0.07, *t*(190) = −3.29, *p* = 0.001). No main effect or interactions involving recipient need were found (*p* ≥ 0.380).

#### Need and reputation trials

The model included fixed effects of the number of stickers shared, combination, whose mood was being rated, their interactions, and children’s age, with participant ID as a random intercept. The model was significantly better than the null (*χ*^2^(16) = 34.39, *p* = 0.005). The change in mood ratings was significantly greater when children rated their own (*M* = 0.44, *SD* = 1.19) compared to the recipients’ mood (*M* = 0.36, *SD* = 1.17) (*b* = 0.44, *SE* = 0.21, *t*(186) = 2.12, *p* = 0.035). The two-way interaction between the number of stickers shared and whose mood was being rated was marginally significant (*b* = −0.05, *SE* = 0.04, *t*(186) =  −1.94, *p* = 0.054). There was no evidence of a main effect of age (*p* = 0.164), nor any significant effects or interactions related to combination (*p* ≥ 0.308).

## Discussion

This study demonstrates, for the first time, that children can consider combinations of recipient characteristics when making costly sharing decisions. While previous research has shown that recipient characteristics such as need^22,38^, merit^45,46^, and generosity^14,27^ influence children’s sharing, these characteristics have rarely been studied in combination (though see ^24,27^). As expected, when children had only information about a potential recipient’s material need, they shared more with needy recipients in costly sharing decisions, consistent with previous findings^22^. In contrast to expectations based on previous developmental trends, there were no age-related changes in children’s sharing behaviour in the need trials in the main analysis. However, when participants who did not pass the training phase were included (see Supplementary Information), an age-related effect emerged, with older children sharing more stickers than younger children. Sharing also increased with age in need and reputation trials. While these results contribute to the growing body of evidence of the role of recipient characteristics on sharing decisions, it is the combination of need and reputation that was of most interest.

When, in addition to information about need, children were provided with testimony about a potential recipient’s reputation for sharing, the change in their sharing between the need trials and the need and reputation trials was greater when the recipient had a positive reputation for sharing compared to a reputation for not sharing. This is consistent with the theory of indirect reciprocity and previous research suggesting that children consider recipients’ prior behaviours such as merit and generosity when they share^22,45–47^. In the need and reputation trials specifically, children shared most when the recipient that was both needy and had a positive reputation, and least when the recipient that was not needy and had a negative reputation. Interestingly, no significant difference was found in the number of stickers shared with recipients who were needy but did not share and those who were not needy but shared. This pattern suggests that sharing was not driven by a preference for only one characteristic, rather it appears that children considered the combination of characteristics.

As well as looking at differences in sharing between the recipients, another point of interest was the fairness of children’s sharing, as older children (6- to 8-years) often show an aversion to inequity, instead demonstrating preferences for fairness by sharing equally with others in costly sharing situations^56,57^. Indeed, the current study showed that when they only knew about the recipients’ need, children shared equally when the recipient was needy and significantly less than half when the recipient was not needy. This suggests that children may have been striving for equity. In this case, both children and the needy recipient started with no resources, so sharing equally ensured that they maintained equal resources. While children were unaware of the precise number of stickers the not needy recipient possessed, they knew that they did have some resources. Thus, sharing less and keeping more for themselves would allow them to approximate equity in terms of total resources, even if the exact amount the recipient had was uncertain. Interestingly, children’s age did not appear to influence these decisions, with even the youngest children following this pattern. This contrasts with previous research which found that older children share more with needy recipients than younger children^22,38^. Later, when children were privy to both the need and the reputation of a potential recipient in the more complex sharing scenarios, they shared significantly more than half with the recipient who had two positive characteristics, equally with recipients who had one positive and one negative characteristic, and significantly less than half with those who had two negative characteristics. If we look at this pattern of sharing through the lens of pursuing fairness, it appears that while children do strive for equity in terms of the number of resources each party has, they also respond to the recipient’s reputation. This is evident in the greater increase in sharing with recipients with a positive reputation for sharing regardless of the recipients’ need. Thus, there is a balance between sharing equally and sharing equitably through indirect reciprocation. These results are in line with other research suggesting that young children have a preference for distributing or sharing equally and yet are able to take recipient characteristics (e.g., merit^45,46^) into account when available to respond equitably.

Together these findings suggest that children consider combinations of recipient characteristics when deciding how much to share, and that this is evident from early in childhood. Although older children shared more, there was no evidence of any interaction with the combinations of characteristics, indicating a consistent pattern of results across ages. Even young children considered both the need and reputation of the recipients when making their sharing decisions. This is somewhat surprising given the developmental trends^22^ previously observed in response to need and reputation individually, as well as the potential challenge of integrating multiple recipient characteristics before making sharing decisions.

This study’s between-participants design meant that each child shared with only one recipient with a specific combination of characteristics, limiting conclusions about preferences when recipients are directly compared. Future research could explore how children allocate resources between a needy recipient with a negative reputation and a not needy recipient with a positive reputation, or how the same child would share across different combinations of characteristics. Prior research found that children allocated resources equally in a non-costly donation scenario^27^ that pit recipients with combinations of kind and non-kind characteristics against one another. Adapting such a paradigm for a costly sharing scenario could address these questions. Another limitation was the absence of ‘reputation only’ trials, which would have allowed for a direct comparison of reputation effects without the influence of need. This omission stemmed from the difficulty of isolating reputation from need in this task. However, previous research suggests children share more with recipients with a positive reputation for sharing^22^. Finally, trial-by-trial data are unavailable as sharing behaviour was recorded in a summary format for each child across each set of trials. Therefore, it was not possible to analyse changes in sharing behaviour over time or in response to decreasing recipient need in this study. Future studies could build on the findings by examining sharing behaviour across individual trials.

The second objective of this study was to investigate whether sharing was motivated by children’s mood ratings—both their own mood and the recipient’s—and how sharing influenced these ratings, focusing on changes from before to after sharing. There was no evidence that mood ratings influenced how much children shared in either set of trials, suggesting that neither their own nor their perception of the recipient’s mood motivated sharing; instead, recipient characteristics were the primary influence. In terms of how sharing behaviour influenced changes in ratings, in need trials, there was a greater positive change in mood rating when children shared less (i.e., kept more stickers) compared to when they shared more (i.e., kept fewer stickers). In other words, children reported being happier after sharing if they kept more for themselves, whereas those who shared more did not experience a similar increase in happiness. In contrast, the change in the recipients’ mood ratings improved as the number of stickers shared increased; children perceived the recipients as being happier when they had received more stickers. The change in the recipients’ mood ratings followed a similar pattern in the need and reputation trials; however, the change in children’s own mood ratings did not vary based on the number of stickers shared. Interestingly, there were no significant interactions or main effects related to recipient characteristics in either set of trials, suggesting that changes in mood ratings were primarily influenced by the amount shared rather than the characteristics of the recipient.

These findings indicate that children monitored the approximate number of stickers both they and the recipient had received, and recognised the potential influence that their sharing could have on the recipient’s mood. However, the initial mood ratings given by children for themselves and the recipient did not predict how much was subsequently shared. Rating recipients as happier when they received more stickers, while not reflecting the same sentiment for themselves, suggests that children may have been motivated by concern for the recipients’ emotional needs, like their response to material need. This aligns with previous research indicating that children’s prosocial behaviour correlates with the recipient’s emotions, suggesting that children may be driven by empathic concern for the recipient rather than their own feelings^52^. That children were not happier when they shared more stands in tension with previous research indicating that children tend to feel happier when they engage in costly giving^50^. There are two potential explanations for the change in children’s own mood ratings. First, children who shared more might not feel happier compared to those who keep more for themselves, because they have fewer stickers for themselves. Alternatively, this pattern may result from a limitation in the mood ratings in this study, as children often rated their mood at the highest level initially, which left limited room for improvement. This ceiling effect may obscure potential increases in happiness associated with sharing more, particularly in the need and reputation trials, as children had already had the opportunity to increase their ratings in the need trials. To address this, future studies could implement a more nuanced emotion scale to capture a broader range of emotional responses. Mood ratings were collected before the first trial and after the last trial in each combination, capturing an overall impression of mood across trials. Thus, it is unclear whether the final ratings reflect the cumulative influence of the six trials or were most strongly influenced by the final trial. Future research could gain a more detailed understanding of sharing affects mood by asking children to rate mood after each trial.

To conclude, when children were provided with information about a potential recipient’s material need and reputation for sharing, they appeared to consider both characteristics in their sharing decisions. They were most generous when the recipient was both needy and had a positive reputation, least generous when the recipient was not needy and had a negative reputation, and equally generous when the recipient displayed one positive and one negative characteristic. Contrary to prior research, there was no evidence suggesting that younger and older children prioritised different recipient characteristics or employed different sharing strategies, indicating that even young children strive for equity in their sharing decisions. Additionally, children demonstrated an awareness of the impact of their choices on the recipient’s mood, shown by the greater change in their ratings of the recipient’s mood when more stickers were shared. Overall, these findings highlight children’s ability to integrate multiple recipient characteristics in their sharing decisions from as early as 3 to 4 years old.

## Method

### Participants

Participants were 186 three- to nine-year-old children (99 females; *M* age = 85.5 months, *SD* = 20.2 months, range = 44 to 118 months) recruited from nurseries and schools serving a broad socioeconomic area in Scotland, UK. Thirteen additional children were tested but excluded due to researcher error (*n* = 4) or not progressing beyond the training phase (*n* = 9).

### Ethical approval

Ethical approval was granted by the Psychology Ethics Committee and the General University Ethics Panel (approval # GUEP10390) at the University of Stirling. Research was conducted in accordance with the British Psychological Society Code of Human Research Ethics guidelines^58^ and informed written consent was obtained from each child’s parent/legal guardian prior to participation.

### Materials

**Fig. 3:**
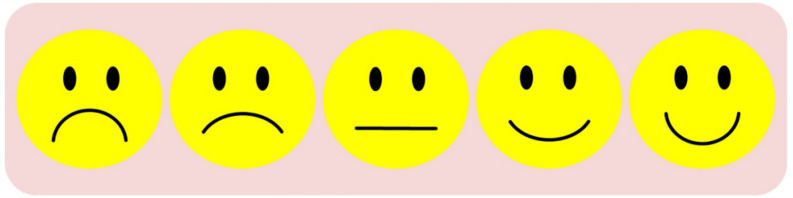
Mood chart used by participants to identify their own and the recipients’ mood.

Five virtual characters (an adult female and two characters of each gender) were presented as potential recipients in the modified dictator game on an Apple iPad. As in previous studies^12,22^, characters were age and gender matched to participants. Stickers were used for sharing and to indicate material need, with transparent bowls used to store characters and participants stickers. A mood chart (Fig. 3) comprised five faces ranging from very unhappy (−2) to very happy (2), the middle face was neutral (0). The order of the mood chart was reversed for half of the participants.

### Procedure

Children were tested individually in a quiet space at their school or nursery by the same female experimenter, who sat next to them. Using a between-subjects design, children were assigned to one of four combinations that manipulated the potential recipients’ material need (needy vs. not needy) and reputation for sharing (sharing vs. not sharing) across two sets of six trials. Both combinations were presented in a single testing session. The first set of trials manipulated material need only, while the second set additionally manipulated reputation, with the recipient’s need matching that of the need trials. The four combinations were: needy and sharing (*n* = 51), not needy and sharing (*n* = 47), needy and not sharing (*n* = 44), and not needy and not sharing (*n* = 44). All children completed the need trials before the need and reputation trials. This order was chosen to mitigate potential carryover effects, as need was manipulated in both sets of trials, while reputation was manipulated only in the second set. Sharing was measured using a modified dictator game^33^, and children kept the stickers they allocated to themselves.

#### Training

Before the two sets of trials, children completed two stages of training. Stage 1: Mood chart. (a) Correctly identify the emotions depicted by each face, (b) use the mood chart appropriately to show how they feel when eating their favourite and least favourite food. Stage 1 was repeated if children did not pass both tasks. Stage 2: Dictator game. The experimenter introduced the adult character on the iPad and positioned a bowl in front of the character and a bowl in front of the child. Using the mood chart, the child was asked to indicate their own feelings and the character’s feelings, both before and after the sharing task. Three identical stickers were placed in front of the child and the experimenter gave them three options: (1) keep all the stickers, (2) give all the stickers to the character, (3) share the stickers. The child could then distribute the stickers into their own bowl and the character’s bowl. If they did not share any of the first three stickers with the character, a second trial with a pair of stickers was conducted. Children who did not allocate any of the five stickers to the character were excluded from analyses. However, including these participants did not alter the overall pattern of results (see Supplementary Information).

#### Need trials

The experimenter introduced the child to a new character and drew attention to the contents of the character’s bowl. Children in the needy combinations were told that the character *“hasn’t got any stickers*,” while children in the not needy combinations were informed that the character *“already has some stickers.”* The child could see that the character’s bowl was empty in the needy combinations or contained some stickers in the not needy combinations, though the exact number of stickers was not visible. The child was then asked to indicate their own feelings and the character’s feelings using the mood chart (Fig. 3); this was repeated after the sixth trial to measure any changes in mood. The experimenter explained that the child would be making more choices about stickers and reminded them of the three options: (1) keep all the stickers, (2) give all the stickers to the character, (3) share the stickers. Over six trials with the same character, the child was presented with three pairs and three trios of stickers in a random order, resulting in a total of 15 stickers available for sharing. Varying the number of stickers per trial created opportunities for equal distribution between the child and the potential recipient, and for trials where equal distribution was not possible, accounting for previous findings that children tend to share equally when the number of resources match the number of people^47^.

#### Need and reputation trials

Six need and reputation trials followed the same procedure as the need trials with a new character, except that children were provided with additional information regarding the character’s reputation for sharing. Children in the sharing combinations were told that the character *“shares with his/her friends at school,”* while those in the not-sharing combinations were told that the character *“does not share with his/her friends at school.”* The experimenter then drew the child’s attention to the new character’s bowl, which matched the initial material need of the character in the need trials; specifically, the new character’s bowl was empty in the needy combinations and contained stickers in the not needy combinations.

### Statistical analyses

Analyses were performed using R^59^, with linear regressions performed using the *stats* package and linear mixed effects models (LMMs) performed using the *lme4* package^60^. Where specified as fixed effects the following variables were sum coded: recipient need (not needy as − 1, needy as 1), combination, whose mood was being rated (recipient as − 1, child as 1). Age was centred and scaled to measure thousands of days. All t-tests and binomial tests are two-tailed.* P*-values less than 0.05 were accepted as statistically significant. Partial eta squared effect sizes for type-III ANOVA tables were calculated for the linear regressions using the *effectsize* package^61^. Post hoc analyses were carried out using estimated marginal means of linear trends with the *emmeans* package^62^. Post hoc results are presented on the log odds ratio scale.

## Supplementary Information

Supplementary Information.

## Data Availability

The data and analysis script are provided on OSF [https://osf.io/9aygb/].
