# Supplementary material for: Children consider others’ need and reputation in costly sharing decisions
Source: Sci Rep. 2025 Feb 27;15:7103. doi: 10.1038/s41598-025-91648-y (PMC11868495; doi:10.1038/s41598-025-91648-y)
Supplement: Supplementary file 1 — Supplementary Information. [file 41598_2025_91648_MOESM1_ESM.pdf]

Children consider others' need and reputation in costly sharing decisions

Supplementary Information

Kirsten H Blakey

Results

The effect of recipient characteristics on sharing

These results include seven of the nine participants who were excluded for not passing the training phase; the remaining two are still excluded due to missing data. Therefore, the sample included 193 three- to nine-year-old children (100 females; *M* age = 84.6 months, *SD* = 20.4 months, range = 44 to 118 months). The additional participants were in the not needy and sharing combination (*n* = 3) and the not needy and not sharing combination (*n* = 4).

Need trials

The linear model examining children's sharing choices in response to recipients' need was significantly better than the null equivalent ($\chi^2(3) = 174.8$, $p < .001$). There was no evidence of a significant interaction between recipient need and age ($p = .801$). Sharing increased significantly with age ($b = 0.46$, $SE = 0.19$, $t(189) = 2.46$, $p = .015$, $\eta^2_p = .03$), and a significant main effect of recipient need ($b = 0.91$, $SE = 0.11$, $t(189) = 7.97$, $p < .001$, $\eta^2_p = .25$) indicated that children shared significantly more with needy recipients ($M = 7.68$, $SD = 1.23$) than with not needy recipients ($M = 5.87$, $SD = 1.91$) (Fig. S1a).

Comparing the number of stickers shared to equal sharing using one-sample t-tests showed that children shared significantly less than half when the recipient was not needy, $t(97) = –8.47$, $p < .001$, but, shared equally when the recipient was needy ($p = .148$).

The difference between need trials and need and reputation trials

The change in the number of stickers shared between the need and reputation trials and the need trials (taken as a baseline) was calculated for each participant. A two-sample t-test revealed a significantly greater increase in children's sharing scores when the recipient had a reputation for sharing ($M = 0.99$, $SD = 2.01$) compared to when the recipient had a reputation not sharing ($M = 0.33$, $SD = 2.22$), $t(184.14) = 2.17$, $p = .032$.

Need and reputation trials

The linear model examining children's sharing choices in response to recipients' need and reputation was significantly better than the null equivalent ($\chi^2(7) = 285.76$, $p < .001$). There was no evidence of an interaction between combination and age ($p = .240$), though sharing did

increase significantly with age (b = 1.07, SE = 0.28, t(185) = 3.87, p < .001, η^2_p = .07). There was a significant main effect of combination, F(3, 185) = 10.66, p < .001, η^2_p = .15 (Fig. S1b). Post hoc pairwise comparisons between combinations showed that children shared significantly more with the needy sharing recipient than the not needy sharing recipient (b = 1.64, SE = 0.47, t(185) = 3.46, p = .004) or the not needy not sharing recipient (b = 2.53, SE = 0.48, t(185) = 5.28, p < .001), and significantly more with the needy not sharing recipient over the not needy not sharing recipient (b = 1.85, SE = 0.50, t(185) = 3.73, p = .001). There were no significant differences between the two needy recipients (p = .514), the two not needy recipients (p = .254), or the needy not sharing and not needy sharing recipients (p = .208).

One-sample t-tests compared children's sharing to sharing equally for each combination of recipient characteristics. Children shared significantly more than half with the needy sharing recipient (M = 8.63, SD = 2.24, t(50) = 3.59, p < .001), equally with the needy not sharing recipient (M = 7.95, SD = 2.36, p = .209) and not needy sharing recipient (M = 7.04, SD = 2.45, p = .190), and significantly less than half with the not needy not sharing recipient (M = 6.10, SD = 2.81, t(47) = –2.54, p = .001).

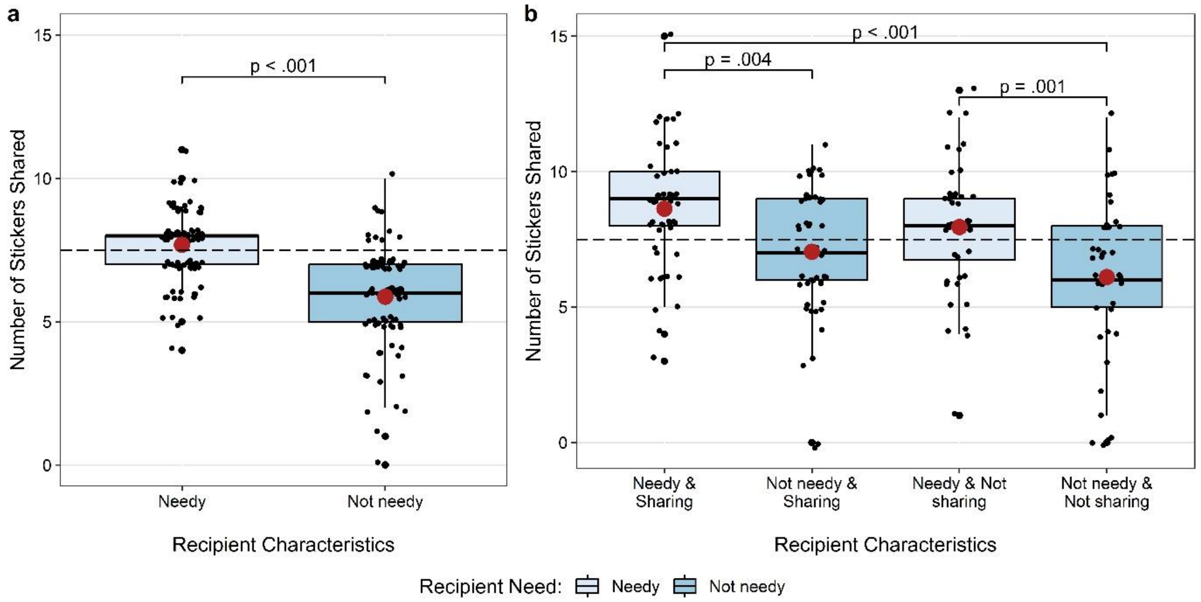

Figure S1. Mean number of stickers shared across the six trials (out of 15) in **a.** the need trials and **b.** the need and reputation trials. Small points show individual sharing scores, and large red points indicate group means. Brackets denote differences between groups. The dashed line represents equal sharing.

The effect of mood rating on sharing

Need trials

To assess whether children's sharing choices were affected by recipients' material need, or the mood ratings children gave for themselves and the recipient, a linear model was constructed with the number of stickers shared in the need trials as the dependent variable and fixed effects of recipient need, initial own mood, initial recipient mood, their interactions, and age. The model was significantly better than the null model ($\chi^2(8) = 123.68$, $p < .001$). There was no evidence of any interactions nor main effects involving mood ratings ($p \geq .202$). The main effects of age and recipient need were the same as in the original model.

Need and reputation trials

To assess whether children's sharing choices were affected by the combinations of recipient characteristics, or the mood ratings children gave for themselves and the recipient, a linear model was constructed with the number of stickers shared in the need trials as the dependent variable and fixed effects of combination of recipient characteristics, initial own mood, initial recipient mood, their interactions, and age. The model was significantly better than the null model ($\chi^2(16) = 161.86$, $p = .015$). There was no evidence of any interactions nor main effects involving mood ratings ($p \geq .476$). The main effects of age and combination were the same as in the original model.
